# Faecal Immunochemical Test (FIT) Sensitivity; A Five Year Audit

**DOI:** 10.3389/bjbs.2024.12862

**Published:** 2024-05-29

**Authors:** Eddie Cole, Deepa Narayanan, Ree Nee Tiam, John Shepherd, Mark O. R. Hajjawi

**Affiliations:** ^1^ Blood Sciences Department, York and Scarborough Teaching Hospitals NHS Foundation Trust, York, United Kingdom; ^2^ Clinical Biochemistry Department, Hull University Teaching Hospitals NHS Trust, Hull, United Kingdom; ^3^ Cellular Pathology Department, Hull University Teaching Hospitals NHS Trust, Hull, United Kingdom

**Keywords:** colorectal cancer, FIT, faecal immunochemical test, sensitivity, bowel cancer, COVID-19, SARS-CoV-2, intestine

## Abstract

**Introduction:** Colorectal cancer has a high prevalence and mortality rate in the United Kingdom. Cancerous colorectal lesions often bleed into the gastrointestinal lumen. The faecal immunochemical test (FIT) detects haemoglobin (Hb) in the faeces of patients and is used as a first line test in the diagnosis of colorectal cancer.

**Materials and Methods:** A retrospective audit of all FIT performed and all colorectal cancers diagnosed in the Hull and East Riding of Yorkshire counties of the United Kingdom (population approximately 609,300) between 2018 and 2022 was conducted. FIT were performed using a HM-JACKarc analyser from Kyowa medical. The predominant symptom suggestive of colorectal cancer which prompted the FIT was recorded. Colorectal cancer was diagnosed using the gold standard of histological biopsy following colonoscopy.

**Results:** Between 2018 and 2022, 56,202 FIT were performed on symptomatic patients. Follow on testing identified 1,511 with colorectal cancer. Of these people, only 450 people with a confirmed colorectal cancer had a FIT within the 12 months preceding their diagnosis. Of these 450 FIT results, 36 had a concentration of <10 μg/g and may be considered to be a false negative. The sensitivity of FIT in the patients identified was 92.00%. The most common reason stated by the clinician for a FIT being performed in patients with colorectal cancer was a change in bowel habits, followed by iron deficient anaemia. The number of patients diagnosed with colorectal cancer decreased in 2020, but increased significantly in 2021.

**Discussion:** This study shows that 8.00% of people diagnosed with colorectal cancer in the Hull and East Riding of Yorkshire regions had a negative FIT. This study also shows that the SARS-CoV-2 pandemic affected the number of people diagnosed with colorectal cancer, and therefore skews the prevalence and pre-test probability of a positive test. There are many reasons why a FIT could produce a false negative result, the most likely being biological factors affecting the stability of haemoglobin within the gastrointestinal tract, or pre-analytical factors influencing faecal sampling preventing the detection of haemoglobin. Some colorectal lesions do not protrude into the gastrointestinal lumen and are less likely to bleed.

**Conclusion:** This is the first study showing data from outside of a structured clinical trial and provides the largest study to date showing the sensitivity of FIT in a routine clinical setting. This study also provides evidence for the impact COVID-19 had on the rate of colorectal cancer diagnosis.

## Introduction

Colorectal cancer is the fourth most common cancer in the United Kingdom [1]. This accounts for more than 40,000 new cases and almost 17,000 deaths [1]. Colorectal cancer also poses a significant financial burden to the United Kingdom, in 2020 it was reported that colorectal cancer cost the UK economy £1.7 billion a year [2]. This cost is a combination of the direct cost from the sum of all healthcare provided and indirect cost from people of working age who are on unable to work, forced into early retirement, or do not survive.

Blood in the stool is a common symptom of colorectal cancer [3]. The Faecal Immunochemical Test (FIT) is a laboratory investigation used to detect haemoglobin (Hb) in faeces, even when the blood may not be visible [3]. The National Institute for Health and Care Excellence (NICE) published its DG30 guidelines for “quantitative faecal immunochemical tests to guide referral for colorectal cancer in primary care” in 2017 [4]. These guidelines state that FIT should be used in patients with a low pre-test probability, but symptomatic of colorectal cancer. It advises the use of FIT for triaging patients before a colonoscopy is performed and that patients with a positive FIT result should be given urgent priority. In 2023, following a publication by D’Souza et al [5], NICE produced the DG56 guidelines recommending that patients with both high risk and low risk symptoms, and therefore both a high and low pre-test probability for cancer should have a FIT test performed.

A number of large clinical trials have reported that FIT has a very high diagnostic sensitivity. In some of these trials, FIT has been reported to have a sensitivity of: 97% (D’Souza et al [5]), 100% (Godber et al [6]), >99% (Ng et al [7]), 100% (Westwood et al [8]), and 100% (McDonald et al [9]). Therefore, a negative result is believed to provide assurance that a person does not have a lower gastrointestinal cancer.

This audit reviewed the diagnostic sensitivity of FIT in all patients diagnosed with colorectal cancer in Hull and East Yorkshire over a 5 year period.

## Materials and Methods

We performed a search of all patient records stored on the Hull University Teaching Hospitals laboratory information management system (LIMS), (Labcentre, Clinisys, Tucson, United States). We identified all patients who had colorectal cancer diagnosed between 2018 and 2022. We searched for patients using the SNOMED codes shown in Appendix 1.

The current gold standard for the diagnosis of colorectal cancer was used. All patients with suspected colorectal cancer had a colonoscopy performed and a biopsy taken. Histological examination of the biopsy sample confirmed the diagnosis of cancer.

Colonoscopy and biopsies were performed by Hull University Teaching Hospitals NHS Trust. Macroscopic and microscopic examination of all biopsy samples was performed. Biopsy specimens taken from the gastrointestinal tract were fixed in neutral buffered formalin for 24 h, resections were fixed for 2–3 days. After dissection, specimens were dehydrated using alcohol, then xylene, and finally embedded in paraffin wax, all using an automated Leica Peloris rapid tissue processor (Milton Keynes, United Kingdom). A Leica Rotary microtome was used to section the embedded tissue samples and the sections were then stained by a Dako (California, United States) automated haematoxylin and eosin stainer. All specimens were analysed by an NHS Consultant Histopathologist as part of routine care.

FIT results, when available, were obtained from the LIMS for all patients with a confirmed diagnosis of colorectal cancer. FIT performed up to 1 year prior to the biopsy were included. FIT performed after the biopsy or greater than 1 year before the biopsy were excluded.

FIT testing was performed within the UKAS ISO 15189 accredited Pathology Laboratory at Hull University Teaching Hospitals using a HM-JACKarc analyser (Kyowa medical, Japan). Calibration and quality control materials were provided by Alpha labs (Hampshire, United Kingdom). Patients collected their own specimen into faecal collection devices (Alpha labs, United Kingdom) containing stabilising buffers to prevent sample degradation [10]. Polyclonal antibodies specific to the globin fraction of Hb bind to any Hb present in the specimen resulting in a turbidimetric change proportional to the concentration. A cut-off value of 10 μg/g of haemoglobin in faeces was regarded as a positive result.

Along with all FIT, the clinician recorded the primary presenting symptom which prompted the suspicion of colorectal cancer. One of five symptoms, all linked to the NICE DG30 guidelines were recorded: unexplained abdominal pain [I], unexplained weight loss [II], changes in bowel habit [III], iron deficient anaemia [IV], or anaemia in the absence of iron deficiency [V].

This is an audit of patient outcomes during routine clinical care, all results generated were part of the patient’s standard treatment. This study was a clinical audit approved by Hull University Teaching Hospitals NHS Trust, reference number BIOC/SE/2024-25/01.

## Results

### FIT Testing and Patient Demographics

**FIGURE 1:**
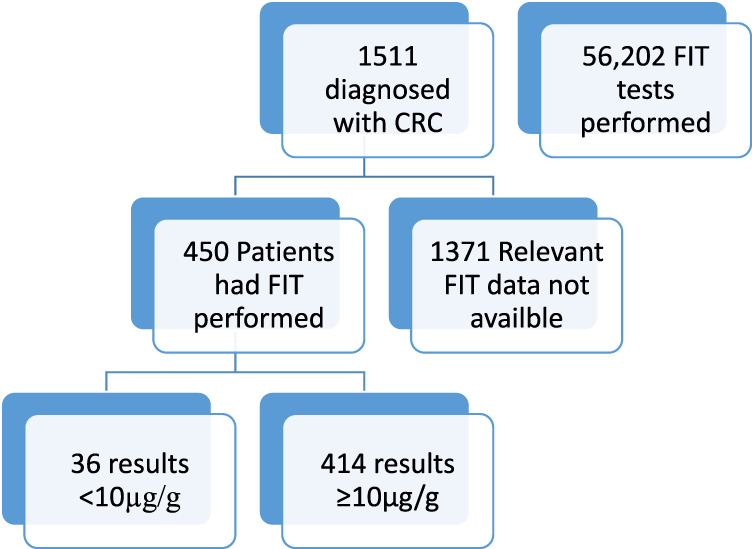
Figure shows total number of patients included and excluded in this study and the availability of FIT data.

The Pathology Laboratory at Hull University Teaching Hospitals performed 56,202 FIT tests in the 5 year period between 2018 and 2022. Of these tests, 41,914 results were <10ug/g and therefore negative and 2009 results were >400 ug/g; this is positive and above the measurement range of the instrument. The remaining specimens which produced a reportable result, the mean result was 67 ug/g. During this time period 1,511 patients were diagnosed with colorectal cancer by colonoscopy and histology. Demographics of the people diagnosed with cancer where as follows; 896 male (59.3%), 615 female (40.7%). The age range of the patients was from 24 to 97, median age 70 years old (IQR 62–77). See Figure 1.

### FIT Sensitivity

**FIGURE 2:**
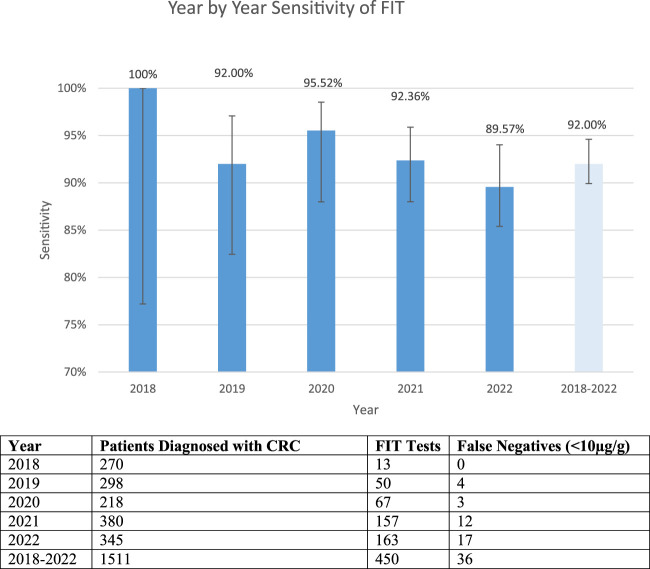
False negative fit results by year and cumulatively. The table shows the total number of patients diagnosed with CRC, FIT tests available on those patients and the number of false negative tests. The graph shows the sensitivity year by year and total (confidence intervals 2018: 77.19%–100%, 2019: 82.45%–97.08%, 2020: 88.14%–95.89%, 2021: 88.00%–95.89%, 2022: 85.40%–94.02% 2018–2022: 89.92%–94.6%).

Of the 1,511 patients diagnosed with colorectal cancer, only 450 had a FIT performed. Of these patients, it was found that 36 patients with colorectal cancer had a false negative FIT result, giving a sensitivity of 92.00%. Figure 2 shows a breakdown of the false negative FIT rate by year. It can be seen that there is a post COVID-19 pandemic rise in the number of patient’s diagnosed with colorectal cancer.

The 36 patients identified includes 22 male (61%) and 14 female (39%). The median and mean ages were 72 and 71.9 years respectively. Blood haemoglobin results pre-admission where reviewed where possible. The median male and female blood haemoglobin results were 127 g/L and 120 g/L, respectively.

Of these 36 patients with FIT negative colorectal cancer, 6 had a histological report that described the removed mass as sessile, that is flat and not protruding into the lumen of the gastrointestinal tract.

### Presenting Symptoms

Table 1 shows the most common reasons why a patient’s NHS General Practitioner requested a FIT. Data was reported for 387 patients who were later diagnosed with colorectal cancer. It can be seen that approximately half of all patients (51.4%) with confirmed colorectal cancer were reported to have a change in bowel habit, and one-fifth (22.5%) were reported to have iron deficient anaemia as the symptom that prompted a FIT.

**TABLE 1:** Clinical reason for performing FIT linked to NICE guidelines. Reason for testing was recorded for 387 patients.

Finding	Frequency	Percentage (%)
Changes in bowel habit	199	51.4
Iron deficient anaemia	87	22.5
Unexplained abdominal pain	72	18.6
Unexplained weight loss	39	10.1
Anaemia in the absence of iron deficiency	27	7.0

Note: Not all GPs submitted a reason for performed the FIT and some GPs identified more than one reason, therefore total is >100%.

## Discussion

This manuscript is the first report on the sensitivity of FIT in a routine clinical setting. Our audit of FIT used under real world conditions, outside of a trial, shows that false negative results are rare but do occur. Our finding that the sensitivity, when using a cut-off value of 10 μg/g, is 92.00%. This finding is in agreement with the reported findings of the clinical trials of Mowat et al [11] 86.7%, Chapman et al [12] 87.5%, Vieto et al [13] 90.8% and Shaukat et al [14] 91%. Using a cut off of <15 μg/g, Katsoula et al [15] reported a sensitivity of 93%, again this is similar to our findings.

Our finding show that a negative FIT result cannot rule out colorectal cancer. It is now commonly adopted clinical practice for a negative FIT result to be used to triage and downgrade a patient’s need for an urgent colonoscopy [16]. The Association for Coloproctology of Great Britain and Ireland suggest that FIT alone is not to be used to exclude a referral [17]. The data we have presented here support this recommendation further. Although it should be noted that the 36 false negative results identified in patients with confirmed cancer equate to 0.06% of all FIT tests performed in this time period. Therefore, the clinician should ensure that safety netting is in place for their patients, but continue to have confidence that a negative result is likely to be correct.

A change in bowel habit was the most common reason for suspecting colorectal cancer in this patient group. It was more than twice as frequent as the next most common symptom and the leading cause for a clinician to request a FIT test in a patient. The second most commonly reported symptom in these patients with confirmed colorectal cancer was iron deficient anaemia. There is a well-established relationship between iron deficient anaemia and colorectal cancer [18].

It was noteworthy that there were fewer patients diagnosed with colorectal cancer in 2020 than in the preceding 2 years. Also of note was the 28% increase in the number of people diagnosed in 2021 with colorectal cancer than any of the preceding 3 years. We suspect this is due to the COVID-19 pandemic, the UK wide lockdown, and reluctance of people to seek help during this period. It would stand to reason that this is to catch up from those missed in the previous year.

If COVID-19 resulted in fewer people seeking help for their medical conditions this may have resulted in a higher prevalence of undiagnosed cancer in the population. This could have led to a higher pre-test probability of cancer, which in turn may have increased the likelihood of a positive FIT test. The impact of this on our study is unknown.

In our patient population, of the 450 patients who had cancer, 36 had a false negative FIT result. There are many reported factors that could affect the diagnostic accuracy of FIT. Sampling of the faecal sample has been reported to be one of the main sources of erroneous results. It has been hypothesised that if a patient takes a sample from a single point of the faeces or towards the centre of the faeces then this may not give a true reflection of the haemoglobin concentration across the full surface of the faeces [19, 20]. A harmonised procedure for FIT specimen collection has been suggested by a number of previous studies Benton et al [19], Godber et al [20] and Fraser [21].

Another explanation which may affect the diagnostic accuracy of the FIT test is the morphology of the colorectal lesion. Some patients have a flat lesion that does not protrude into the gastrointestinal lumen. Dysplasia which arises from sessile serrated polyps known as sessile serrated adenoma or sessile serrated lesion are much less likely to bleed than some of the more common lesions. Patients with a flat lesion are also more likely to be asymptomatic [22, 23]. Of 36 patients identified in our audit 6 patient’s histological reports mentioned sessile masses. FIT is designed to detect haemoglobin in the faeces, it may have to be accepted that these patients will not produce a true positive result because of the nature of their pathology.

Fraser et al [24] suggested that the slower transition time through the digestive tract in women accounts for a higher probability of a false negative FIT in female vs. male patients. In women if the lesion is higher up the gastrointestinal tract the haemoglobin is potentially more likely to deteriorate before it is eliminated and sampled by the patient. Our data found a similar 60/40 male-female ratio in both total patients diagnosed with colorectal cancer and patients with a false negative FIT result. However we recognise that with only 36 false negative patients this is not a large enough number to give certainty that there is not a higher false negative rate in female patients.

A weakness of our study is that it focuses on only those patients with a known diagnosis of colorectal cancer. The outcome of all 56,202 people who had a FIT test during the 5 year period is not known. Potential further work on FIT could look at those biopsies where colorectal cancer was not identified and the use of FIT to detect other pathologies. Therefore our study does not highlight and review the full utility of the FIT test. A negative result is just as important as a positive result in the differential diagnosis of a patient.

## Summary Table

### What Is Known About the Subject

• Colorectal cancer accounts for a significant proportion of UK cancer incidence and deaths.
• Blood in the stool is a common finding in colorectal cancer.
• FIT is a test designed to identify blood in the stool of symptomatic colorectal cancer patients.

### What This Papers Adds

• The negative predictive value of FIT in clinical practice may not be as high as initially reported in clinical trials.
• Some insight into the impact of the COVID-19 pandemic on the diagnosis of colorectal cancer.
• Consideration of the accuracy and limitations of FIT.

## Concluding Statement

This work represents an advance in biomedical science because it highlights the utility of a test in practice, and sheds light on the impact of COVID-19 on cancer diagnosis rates.

## Data Availability

The raw data supporting the conclusion of this article will be made available by the authors, without undue reservation.

## Appendix 1 | SNOMED codes identified in colorectal cancer patients with description.

**Table:**

SNOMED codes	SNOMED description
P206021	Colon neoplasm screening
T67000	Colon, NOS
T67100	Cecum
T67200	Ascending colon
T67400	Transverse colon
T67600	Descending colon
T67700	Sigmoid colon
T68000	Rectum, NOS
T69000	Anus, NOS
